# Supplementary material for: Hepatitis B Core Protein Is Post-Translationally Modified through K29-Linked Ubiquitination
Source: Cells. 2020 Nov 26;9(12):2547. doi: 10.3390/cells9122547 (PMC7760836; doi:10.3390/cells9122547)
Supplement: Supplementary file 1 [file cells-09-02547-s001.pdf]

cells

MDPI

# Supplementary Materials

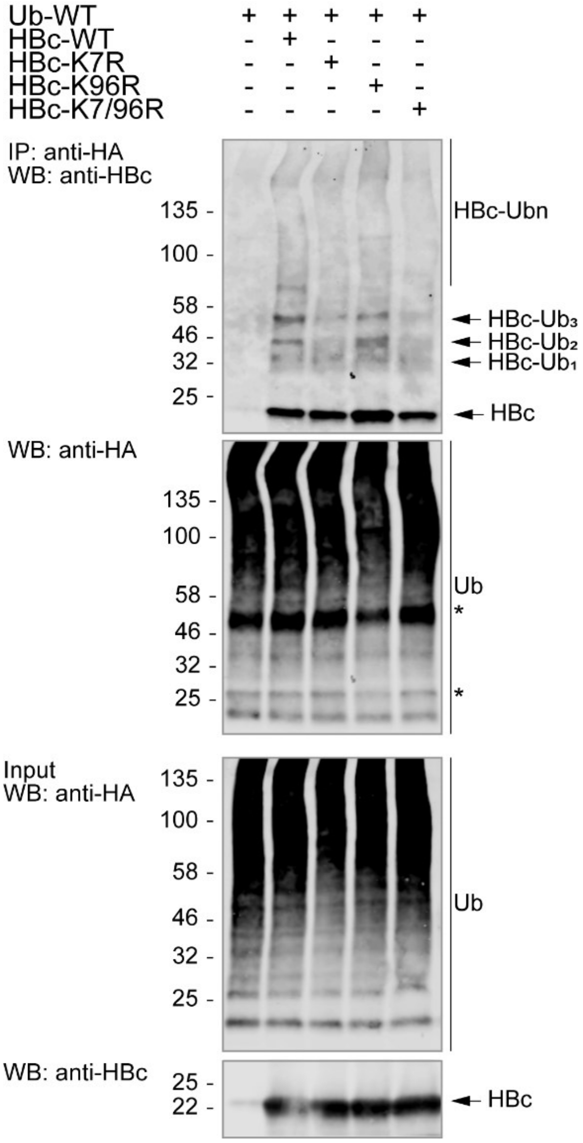


**Figure S1.** HBc without tag is ubiquitinated in the same manner as FLAG-tagged HBc. For transient co-transfection, HBc without tag and its mutants (HBc-K7R, HBc-K96R, HBc-K7/96R) and HA-Ub WT were used. Forty-eight hours post-transfection, ubiquitin was precipitated from precleared cell lysates using anti-HA magnetic beads. Immunoprecipitated complexes were analyzed by Western blot using an LI-COR Odyssey CLx system and Image Studio Lite Software.

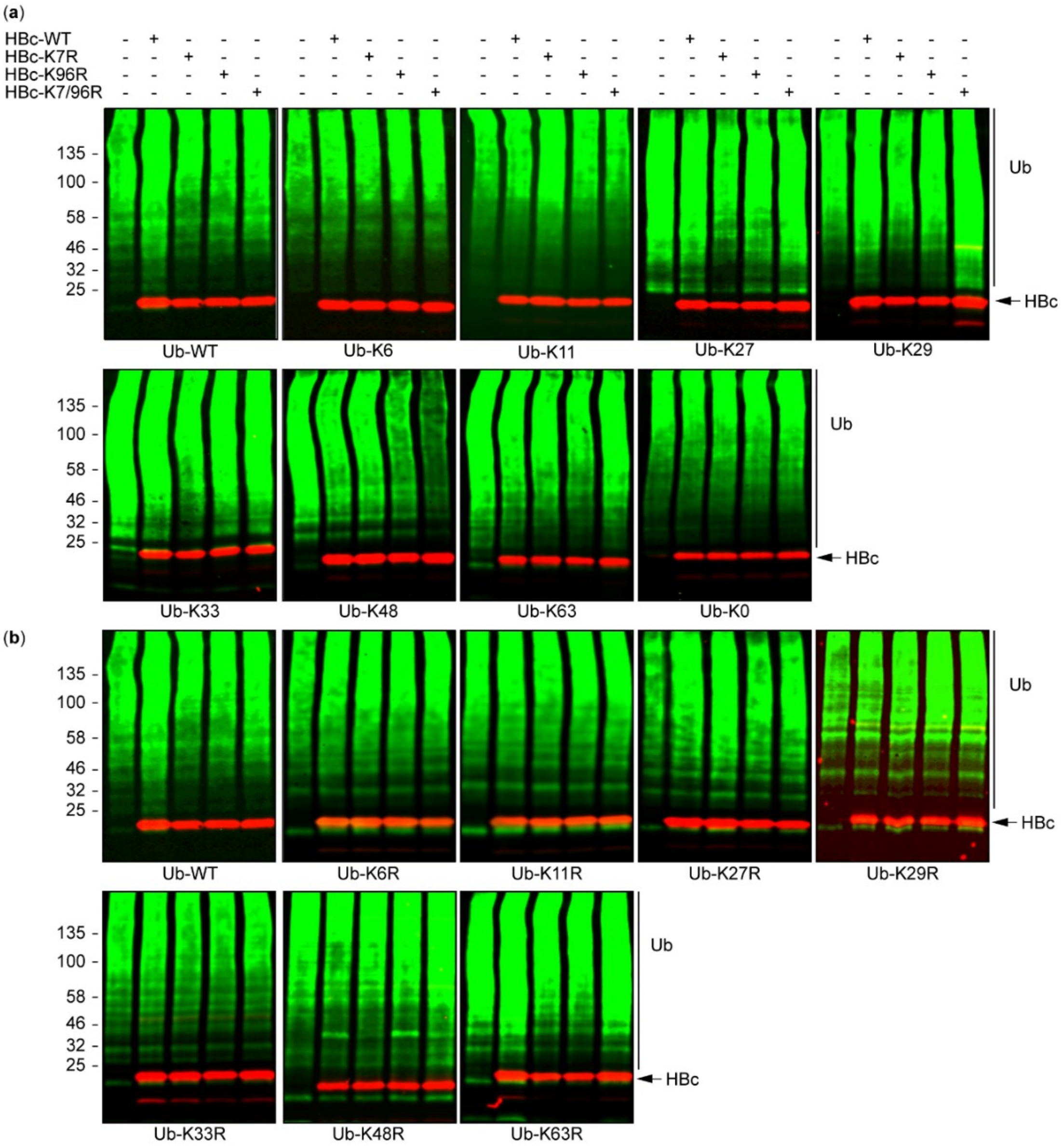


**Figure S2.** Expression of HBc variants and different ubiquitin variants: (**a**) Ubiquitin single K mutants and lysine-free ubiquitin K0; and (**b**) ubiquitin K-to-R mutants (WB: Input 10 μg of total protein/track). Huh7 cells were transiently co-transfected with one of ubiquitin variants and one of the HBc variants. Protein expression was analyzed by Western blot. The rabbit antibody (IRDye 800CW) against HA-tag was used for ubiquitin detection and the mouse antibody (IRDye 680RD) against FLAG-tag was used for HBc detection.

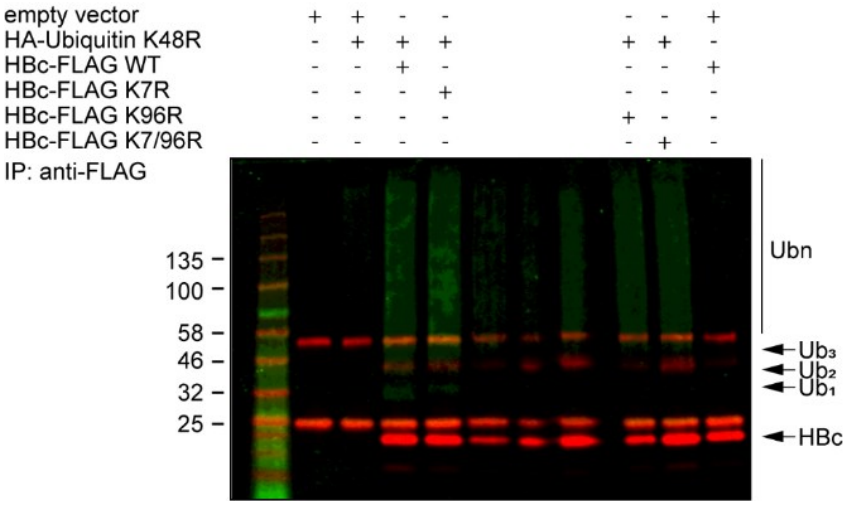


**Figure S3.** Ubiquitination of HBc by the K48 ubiquitin variant. Original figure of Western blot. Immunoprecipitated complexes were analyzed by Western blot. The samples were loaded into lanes 6, 7, and 8 unevenly. The rabbit antibody (IRDye 800CW) against HA-tag was used for ubiquitin detection and the mouse antibody (IRDye 680RD) against FLAG-tag was used for HBc detection.

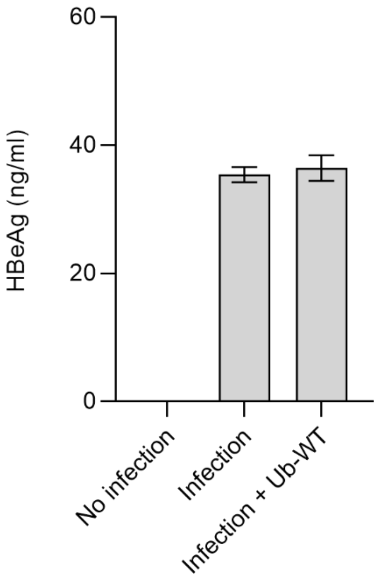


**Figure S4.** Ubiquitin overexpression in HBV-infected cells does not influence an early marker HBeAg of viral replication. Forty-eight hours post-transfection of HBV-infected cells, the medium was harvested from three biological replicates and the quantity of secreted precore antigen (HBeAg) was determined using the ELISA technique. Data is shown as mean ± standard deviation.

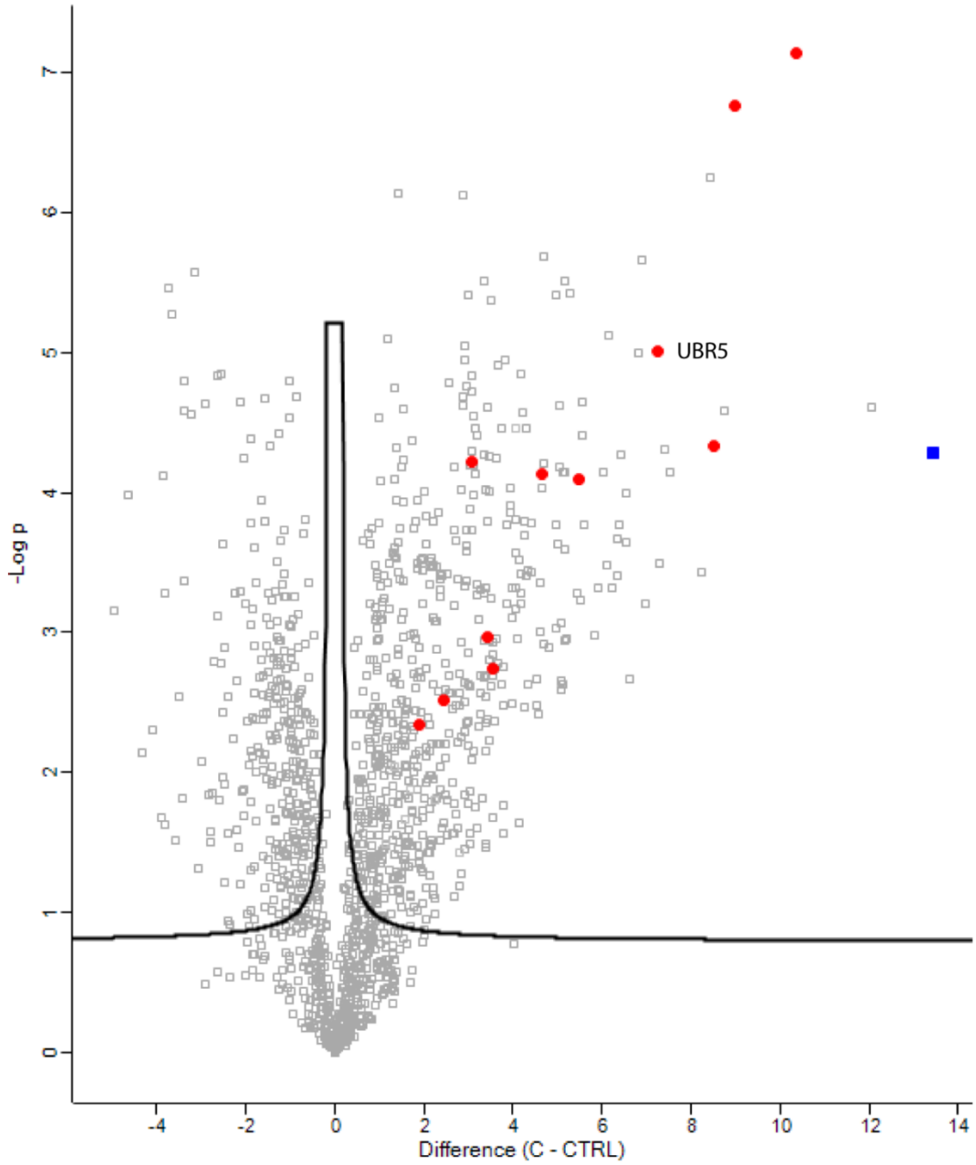


**Figure 5.** Volcano plot of the shotgun LC-MS/MS analysis of proteins co immunoprecipitated with HBc-HA expressed in HEPG2-hNTCP. Differential expression patterns between the HBc-HA sample and negative control sample (CTRL, empty vector) performed in biological triplicates. Red points in the plot represent the E3 ubiquitin-protein ligases with 1.5-fold upregulation with statistical significance $p < 0.01$ (for a detailed list, see Table 2). Blue square represents HBc. E3 ubiquitin-protein ligase, UBR5, known to catalyze K29-linked ubiquitination is marked.

**Table S1.** E3 ubiquitin-protein ligases identified by shotgun LC-MS/MS analysis of proteins precipitated with empty vector, Ub-WT, Ub-K29, and d in Huh7 cells.

| E3 Ubiquitin-Protein Ligase | Ubi-WT/K29/K29R/CTRL # Peptides | Ubi-WT/K29/K29R/CTRL # PSMs |
|---|---|---|
| E3 ubiquitin-protein ligase hectd1 | 32/32/11/0 | 48/52/17/0 |
| E3 ubiquitin-protein ligase Itchy homolog | 14/22/1/0 | 19/33/1/0 |
| E3 ubiquitin-protein ligase UBR5 | 8/2/0/0 | 10/3/0/0 |
| E3 ubiquitin-protein ligase HUWE1 | 5/2/1/0 | 6/3/4/0 |
| E3 ubiquitin-protein ligase RBBP6 | 5/2/7/5 | 6/2/8/7 |
| E3 ubiquitin-protein ligase NEDD4-like | 4/2/0/0 | 5/2/0/0 |
| E3 ubiquitin-protein ligase HECW2 | 3/2/0/0 | 4/2/0/0 |
| E3 ubiquitin-protein ligase NEDD4 | 2/1/0/0 | 2/1/0/0 |
| E3 ubiquitin-protein ligase RNF220 | 2/2/0/0 | 2/2/0/0 |
| NEDD4-like E3 ubiquitin-protein ligase WWP1 | 1/2/0/0 | 2/4/0/0 |
| E3 ubiquitin-protein ligase TRIP12 | 1/2/1/0 | 2/2/1/0 |
| E3 ubiquitin-protein ligase ARIH1 | 1/6/0/0 | 1/7/0/0 |
| E3 ubiquitin-protein ligase TRIM4 | 1/1/1/0 | 1/1/1/0 |
| E3 ubiquitin-protein ligase RNF114 | 1/1/0/0 | 1/1/0/0 |
| E3 ubiquitin-protein ligase RNF123 | 1/1/1/1 | 1/1/1/1 |
| E3 ubiquitin-protein ligase RNF25 | 0/9/0/0 | 0/13/0/0 |
| E3 ubiquitin-protein ligase RNF19A | 0/8/0/0 | 0/12/0/0 |
| E3 ubiquitin-protein ligase RNF5 | 0/1/0/0 | 0/2/0/0 |
| E3 ubiquitin-protein ligase RAD18 | 0/2/0/0 | 0/2/0/0 |
| E3 ubiquitin-protein ligase rififylin | 0/1/0/0 | 0/1/0/0 |
| E3 ubiquitin-protein ligase KCMF1 | 0/2/0/0 | 0/2/0/0 |
| E3 ubiquitin-protein ligase RNF103 | 0/1/0/0 | 0/1/0/0 |
| E3 ubiquitin-protein ligase RNF31 | 0/1/0/0 | 0/2/0/0 |
| E3 ubiquitin-protein ligase mib1 | 0/1/0/0 | 0/1/0/0 |
| NEDD4-like E3 ubiquitin-protein ligase WWP2 | 0/1/0/0 | 0/2/0/0 |
| E3 ubiquitin-protein ligase UBR4 | 0/2/0/0 | 0/2/0/0 |

(Ubi-WT, pRK5-HA-Ubiquitin WT; Ubi-K29, pRK5-HA-Ubiquitin K29; Ubi-K29R, pRK5-HA-Ubiquitin K29R; CTRL, empty vector; # peptides, the number of detected peptide sequences unique to a protein; # PSM, the total number of identified peptide spectra matched for the protein).

**Table S2.** E3 ubiquitin-protein ligases identified by shotgun LC-MS/MS analysis of proteins co-immunoprecipitated with HBc-HA expressed in HEK293T cells.

| E3 Ubiquitin-Protein Ligase | HBc # Peptides Exp1/Exp2 | HBc # PSMs Exp1/Exp2 | CTRL# Peptides Exp1/Exp2 | CTRL # PSMs Exp1/Exp2 |
|---|---|---|---|---|
| E3 ubiquitin-protein ligase MYCBP2 | 78/122 | 133/202 | 16/68 | 23/102 |
| E3 ubiquitin-protein ligase HERC2 | 14/25 | 22/37 | 0/6 | 0/7 |
| E3 ubiquitin-protein ligase TRIM21 | 19/21 | 35/49 | 24/29 | 60/81 |
| E3 ubiquitin-protein ligase Midline-1 | 11/13 | 19/32 | 6/11 | 10/19 |
| Probable E3 ubiquitin-protein ligase HECTD4 | 6/12 | 7/19 | 0/1 | 0/1 |
| E3 ubiquitin-protein ligase RNF213 | 16/10 | 21/14 | 0/4 | 0/5 |
| Probable E3 ubiquitin-protein ligase makorin-2 | 2/9 | 3/14 | 2/7 | 3/11 |
| E3 ubiquitin-protein ligase listerin | 13/9 | 18/14 | 2/5 | 3/9 |
| E3 ubiquitin-protein ligase UBR5 | 6/8 | 10/9 | 3/3 | 3/4 |
| E3 ubiquitin-protein ligase TRIM4 | 7/8 | 7/13 | 6/9 | 7/13 |
| E3 ubiquitin-protein ligase BRE1B | 7/5 | 11/9 | 3/3 | 3/4 |
| E3 ubiquitin-protein ligase HECTD1 | 3/5 | 6/8 | 1/4 | 1/5 |
| E3 ubiquitin-protein ligase RFWD3 | 2/5 | 2/6 | 0/4 | 0/6 |
| E3 ubiquitin-protein ligase CBL OS | 4/4 | 7/7 | 3/4 | 5/9 |
| E3 ubiquitin-protein ligase RNF138 | 4/4 | 6/5 | 2/5 | 3/8 |
| E3 ubiquitin-protein ligase RNF114 | 2/4 | 2/7 | 2/9 | 3/14 |

| | | | | |
|---|---|---|---|---|
| E3 ubiquitin-protein ligase RNF187 | 1/4 | 2/6 | 1/4 | 2/6 |
| E3 ubiquitin-protein ligase LRSAM1 | 2/4 | 2/7 | 0/4 | 0/6 |
| E3 ubiquitin-protein ligase TRIM33 | 1/3 | 1/4 | 1/2 | 1/3 |
| E3 ubiquitin-protein ligase TRIM32 | 1/3 | 2/6 | 0/1 | 0/2 |
| E3 ubiquitin-protein ligase makorin-1 | 0/3 | 0/3 | 0/0 | 0/0 |
| E3 ubiquitin-protein ligase TRIM9 | 7/3 | 8/4 | 5/1 | 6/1 |
| E3 ubiquitin-protein ligase TRIM71 | 2/3 | 3/4 | 0/1 | 0/2 |
| E3 ubiquitin-protein ligase TRIP12 | 14/3 | 15/5 | 2/0 | 3/0 |
| E3 ubiquitin-protein ligase MIB1 | 1/3 | 2/4 | 0/2 | 0/3 |
| E3 ubiquitin-protein ligase CBL-B | 2/2 | 4/4 | 0/2 | 0/2 |
| E3 ubiquitin-protein ligase TRIM56 | 0/2 | 0/2 | 0/0 | 0/0 |
| E3 ubiquitin-protein ligase BRE1A | 4/2 | 7/3 | 2/0 | 3/0 |
| E3 ubiquitin-protein ligase RNF31 | 0/2 | 0/2 | 0/0 | 0/0 |
| RNA-binding E3 ubiquitin-protein ligase MEX3C | 0/2 | 0/3 | 0/1 | 0/2 |
| E3 ubiquitin-protein ligase UHRF1 | 0/2 | 0/3 | 0/0 | 0/0 |
| Probable E3 ubiquitin-protein ligase MID2 | 1/2 | 1/4 | 0/2 | 0/3 |
| E3 ubiquitin-protein ligase Praja-2 | 1/2 | 1/3 | 1/1 | 1/2 |
| E3 ubiquitin-protein ligase SHPRH | 1/2 | 2/3 | 0/0 | 0/0 |
| E3 ubiquitin-protein ligase Praja-1 | 1/1 | 1/1 | 0/0 | 0/0 |
| E3 ubiquitin-protein ligase RNF126 | 0/1 | 0/1 | 0/2 | 0/3 |
| E3 ubiquitin-protein ligase TRIM23 | 0/1 | 0/1 | 0/0 | 0/0 |
| E3 ubiquitin-protein ligase RLIM | 1/1 | 1/1 | 0/0 | 0/0 |
| E3 ubiquitin-protein ligase RING2 | 1/1 | 2/2 | 0/0 | 0/0 |
| E3 ubiquitin-protein ligase ARIH1 | 0/1 | 0/2 | 0/4 | 0/5 |
| NEDD4-like E3 ubiquitin-protein ligase WWP1 | 1/1 | 1/1 | 1/1 | 2/1 |
| E3 ubiquitin-protein ligase NRDP1 | 0/1 | 0/2 | 0/2 | 0/2 |
| E3 ubiquitin-protein ligase RBX1 | 0/1 | 0/2 | 0/1 | 0/1 |

| **E3 Ubiquitin-Protein Ligase** | **HBc # Peptides Exp1/Exp2** | **HBc # PSMs Exp1/Exp2** | **CTRL# Peptides Exp1/Exp2** | **CTRL # PSMs Exp1/Exp2** |
|---|---|---|---|---|
| E3 ubiquitin-protein ligase XIAP | 0/1 | 0/1 | 0/0 | 0/0 |
| E3 ubiquitin-protein ligase TTC3 | 1/1 | 2/1 | 0/0 | 0/0 |
| E3 ubiquitin-protein ligase RNF31 | 0/1 | 0/1 | 0/0 | 0/0 |
| E3 ubiquitin-protein ligase RNF220 | 0/1 | 0/1 | 0/1 | 0/1 |
| E3 ubiquitin-protein ligase HUWE1 | 2/1 | 2/1 | 0/1 | 0/1 |
| Ubiquitin-protein ligase E3C | 0/1 | 0/4 | 0/0 | 0/0 |
| E3 ubiquitin-protein ligase RBBP6 | 2/0 | 2/0 | 0/0 | 0/0 |
| NEDD4-like E3 ubiquitin-protein ligase WWP2 | 2/0 | 2/0 | 1/0 | 2/0 |
| E3 ubiquitin-protein ligase Itchy homolog | 2/0 | 3/0 | 1/0 | 2/0 |
| E3 ubiquitin-protein ligase LNX | 1/0 | 1/0 | 0/0 | 0/0 |
| E3 ubiquitin-protein ligase pellino homolog 3 | 1/0 | 1/0 | 0/0 | 0/0 |
| E3 ubiquitin-protein ligase ZFP91 | 1/0 | 1/0 | 0/0 | 0/0 |
| E3 ubiquitin-protein ligase RING1 | 1/0 | 2/0 | 0/0 | 0/0 |

(HBc, HBc-HA sample; CTRL, negative control (no HBc) sample; # peptides, the number of detected peptide sequences unique to a protein; # PSM, the total number of identified peptide spectra matched for the protein).
